# Direct bone marrow HSC transplantation enhances local engraftment at the expense of systemic engraftment in NSG mice

**DOI:** 10.1038/srep23886

**Published:** 2016-04-11

**Authors:** Kathryn Futrega, William B. Lott, Michael R. Doran

**Affiliations:** 1 Queensland University of Technology (QUT) at the Translational Research Institute (TRI), 37 Kent Street, Brisbane, Queensland, Australia 4102; 2 Mater Medical Research – University of Queensland at the Translational Research Institute (TRI), 37 Kent Street, Brisbane, Queensland, Australia 4102

## Abstract

Direct bone marrow (BM) injection has been proposed as a strategy to bypass homing inefficiencies associated with intravenous (IV) hematopoietic stem cell (HSC) transplantation. Despite physical delivery into the BM cavity, many donor cells are rapidly redistributed by vascular perfusion, perhaps compromising efficacy. Anchoring donor cells to 3-dimensional (3D) multicellular spheroids, formed from mesenchymal stem/stromal cells (MSC) might improve direct BM transplantation. To test this hypothesis, relevant combinations of human umbilical cord blood-derived CD34^+^ cells and BM-derived MSC were transplanted into NOD/SCID gamma (NSG) mice using either IV or intrafemoral (IF) routes. IF transplantation resulted in higher human CD45^+^ and CD34^+^ cell engraftment within injected femurs relative to distal femurs regardless of cell combination, but did not improve overall CD45^+^ engraftment at 8 weeks. Analysis within individual mice revealed that despite engraftment reaching near saturation within the injected femur, engraftment at distal hematopoietic sites including peripheral blood, spleen and non-injected femur, could be poor. Our data suggest that the retention of human HSC within the BM following direct BM injection enhances local chimerism at the expense of systemic chimerism in this xenogeneic model.

Umbilical cord blood (CB)-derived hematopoietic stem cells (HSC) are ideal for use in allogeneic HSC transplantation when a compatible adult donor is unavailable. However, the limited number of HSC per unit of CB delays engraftment and may be associated with graft failure and/or mortality^1^. The limited cell number is compounded by the inefficiency with which the transplanted cells home to the patient’s bone marrow (BM). To overcome cell number and homing limitations, a number of strategies have been trialed including transplantation of multiple CB units, *ex vivo* expansion prior to transplantation, manipulation of the cell graft to enhance homing efficiency, and direct BM injection.

Two recent clinical trials, one using immobilized Notch ligand^2^ and the other using mesenchymal stem/stromal cell (MSC) co-culture^3^, demonstrated that *ex vivo* expansion could increase the CD34^+^ progenitor cell number by 100- or 40-fold, respectively. In both cases the expanded progenitor cells facilitated rapid myeloid reconstitution, but did not contribute to long-term hematopoiesis in the human recipients. Despite significant advancements in the field, strategies that maintain populations of long-term repopulating HSC during extended *ex vivo* expansion remain elusive^4^. Until this barrier is overcome, *ex vivo* expanded CB units must be supplemented with a unit of unmanipulated CB, significantly increasing the total cost of the therapy^4^. This significant cost has motivated investment into potentially more cost-effective strategies such as those that may improve the efficiency by which donor cells home to the recipient’s marrow.

In mouse models^5^ and a phase I clinical trial^6^, a brief 2 hour pre-treatment of CB cells with 16,16-Dimethyl prostaglandin E2 (PGE_2_) significantly enhanced cell homing efficiency. While the total number of CD34^+^ cells that homed to the murine BM was not reported, the frequency of CD34^+^ cells in the murine femurs increased by ~50% when the transplanted cells were pre-treated with PGE_2_. Data from the associated human trial indicated a promising trend in neutrophil and platelet recovery kinetics, and critically, the manipulated cells continued to contribute to hematopoiesis up to the time of publication (27 months)^6^. Similarly, a recent study from Shpall’s group demonstrated that enzymatically fucosylated CB cells had enhanced homing capacity leading to improved clinical outcomes^7^.

Theoretically, direct BM injection should enable bypassing aspects of the homing process and reduce donor cell loss to non-hematopoietic organs. There is evidence in murine models that direct BM transplantation is superior to intravenous transplantation when donor cell numbers are limiting^8^,^9^ or when transplanting cells with impaired homing capacity^10^. However, this has not translated to improvements in human CB transplant outcomes in the clinic^11^. While recent failure to demonstrate benefit in the clinic will likely deter near-term human studies, the conceptual merits of direct BM transplantation coupled with controversial animal model results^8^,^12^,^13^,^14^,^15^,^16^,^17^ could continue to drive research in this area. The most agreed upon limitation in current direct BM injection methods is that donor cells are not necessarily retained within the injected marrow. Legitimizing this concern are studies suggesting that only ~10% of donor cells are physically retained in the injected marrow even 5 minutes post transplantation^17^. Not only does vascular perfusion redistribute injected cells, the proximal delivery of these cells into the BM cavity may itself be insufficient to functionally replace active homing or retention. In studies where the CXCR4 receptor was blocked on donor cells, delivery into the BM did not rescue engraftment capacity^18^. These results suggest that active homing processes are necessary for engraftment, regardless of delivery route. Resolving the debate as to whether failure to retain donor cells within the injected marrow is the limiting factor would require experimentation involving the anchorage of the donor cells within the marrow using a material, which itself does not compromise donor cell function.

Assuming donor cell retention in the injected marrow was a limiting factor, we hypothesized that engraftment following direct BM transplantation could be improved if donor HSC were anchored to multicellular BM-derived mesenchymal stromal cell (MSC) spheroids. Studies suggest that the biological potency of MSC, and specifically their HSC-supportive capacity, is increased when these cells are assembled into 3-dimensional (3D) spheroids^19^,^20^, making MSC spheroids an appropriate biologically active anchor. Herein, we describe a high-throughput microwell platform to manufacture thousands of multicellular spheroids, each formed from approximately 110 MSC and 10 CB-derived CD34^+^ cells. Spheroids formed spontaneously when the CD34^+^ cells and MSC were co-cultured for 24 hours, and the spheroid diameter (<100 μm) was tailored to enable direct BM injection using a fine 27-gauge needle. We used a short culture period (24 hours) to minimize the negative impact of *ex vivo* expansion on long-term HSC engraftment. To test our hypothesis, we compared four transplant conditions in sub-lethally irradiated NOD/SCID gamma (NSG) mice: **(1)** intravenous (IV) injection of CD34^+^ cells, **(2)** intrafemoral (IF) injection of CD34^+^ cells, **(3)** IF injection of CD34^+^ cells and MSC in suspension, and **(4)** IF injection of CD34^+^ cells and MSC organized into 3D spheroids (summarized in Fig. 1).

## Results

### MSC isolation, culture and characterization

Adult BM-derived MSC met the typical criteria defined for MSC (see Supplementary Figure 1) including plastic-adherent, differentiated to adipocytes, osteoblasts and chondrocytes, and expressed surface antigens CD44, CD73, CD90, CD105, CD146, and lacked the expression of hematopoietic markers, CD45, CD34 and HLA-DR.

### Characterization of CD34^+^ and MSC 2D and 3D co-cultures

We characterized both 2D and 3D co-cultures at 24 hours. Figure 2A–D show the 2D co-cultures and Fig. 2E–L show the development of 3D co-cultures. CD34^+^ cells were labeled with CFSE (green) and MSC labeled with CMPTX (red). Figure 2E–I shows the assembly of 3D spheroids consisting of labeled CD34^+^ cells and MSC at time zero in microwells. Figure 2J–L show the 3D spheroids after they had been depleted of loose CD34^+^ cells. Through manual counting we found that on average, 3 CD34^+^ cells were attached to a MSC spheroid, following 24-hours.

### NSG engraftment

Engraftment in the BM, peripheral blood and spleen were characterized via flow cytometry. Supplementary Figure 2 provides an example of the gating strategy used for flow cytometry analysis. Figure 3 displays individual mouse and median hCD45 engraftment observed at 8 weeks post transplant in (A) the peripheral blood, (B) spleen, (C) right & left femurs, and (D) the average of both femurs. When CD34^+^ cells only (no MSC) were delivered via IV or IF injection, the median peripheral blood engraftment was similar in these groups (Fig. 3A). When CD34^+^ cell IF transplants were co-transplanted with a suspension of MSC, there was an incremental reduction (not statistically significant) in peripheral blood engraftment relative to IV or IF transplantation containing CD34^+^ cells only. hCD45 engraftment was significantly lower in the spleen when MSC where co-transplanted with CD34^+^ cells through IF injection (Fig. 3B), relative to IV CD34^+^ transplants. A statistically significant decrease in peripheral blood and spleen engraftment was observed in animals that received IF CD34^+^ cell transplants with 3D MSC spheroids, relative to IF CD34^+^ cells alone. Overall, peripheral blood and spleen engraftment was similar between IF and IV transplant groups receiving CD34^+^ cells alone, while engraftment appeared to be incrementally compromised in IF groups with the addition of MSC in suspension or in spheroids.

We subsequently contrasted hCD45 engraftment between femurs in all animals. Engraftment in left and right femurs did not differ significantly in individual mice following IV human cell transplantation (Fig. 3C). Given the relatively uniform total body irradiation used in the conditioning procedure, uniform BM engraftment following IV transplantation was not surprising. In contrast to IV results, but similar to previous reports^8^,^12^,^17^, IF transplantation resulted in greater hCD45 engraftment within injected right femurs (RF) relative to distal left femurs (LF). The relative human engraftment between femurs in individual mice is shown graphically in Supplementary Figure 3A–D. While hCD45 engraftment was always greater within injected femurs (RF), the BM hCD45 content was not different between experimental groups (groups 1–4) when the injected and distal femur BM hCD45 numbers were averaged (Fig. 3D).

We further evaluated the lineage composition of the hCD45 populations between femurs, peripheral blood and spleen of mice. The relative CD34^+^ progenitor cell content was similar in both femurs following IV transplantation (Fig. 3E). By contrast, the CD34^+^ cell content, was significantly greater in injected femurs than in non-injected femurs following IF transplantation. In a previous study, a similar pattern was interpreted as either the increased retention of progenitor cells within the injected femur or an increased rate of local self-renewal within injected femurs^17^. To determine if other lineage populations were different between femurs of individual animals, we quantified the relative CD19^+^ (B cell), CD33^+^ (myeloid), and CD15^+^ (granulocyte) lineage committed cells in each femur, peripheral blood and spleen (Supplementary Figures 4 and 5). The portion of hCD45 cells that were CD19^+^, CD33^+^, or CD15^+^ was similar in the femurs of mice from all four transplant groups (Supplementary Figure 5). CD15^+^ human cells were rare (≤1% - data not shown) in the peripheral blood and spleen. Relative numbers of human CD33^+^ and CD19^+^ cells found in the peripheral blood and spleen are shown in Supplementary Figure 5. Overall, with the exception of CD34^+^ cell content in the injected and distal femurs of IF animals, human hematopoietic lineage populations were similar across all groups.

The significantly different hCD45^+^ and CD34^+^ progenitor cell content between injected and distal femurs indicated that BM engraftment within individual mice was heterogeneous and dependent on delivery site. To probe this further, the hCD45 engraftment ratio between the injected femur (RF) and distal femur (LF) was calculated (referred to as “RF/LF ratio”). The RF/LF ratio is a measure of the engraftment heterogeneity within a single animal. An RF/LF ratio of 1 represents an equal distribution of hCD45 cells engrafted between femurs, and suggests that engraftment within that individual animal was relatively homogenous. An RF/LF ratio of 10, for example, indicates that the engraftment in the injected femur (RF) was 10-fold greater than in the non-injected femur (LF) and that the engraftment was heterogeneous. The RF/LF ratios were plotted against hCD45 engraftment in peripheral blood, spleen, non-injected distal femur (LF), and injected femur (^*^RF) for each animal (Fig. 4A–D, respectively).

Animals with the highest levels of peripheral blood engraftment (top 15 out of 60 animals) had RF/LF ratios ≤2 (Fig. 4A), suggesting that high levels of engraftment were consistent with more equal distribution of donor cells between femurs. By contrast, lower levels of peripheral blood engraftment were associated with RF/LF ratios >2, suggesting that failure to achieve homogenous BM engraftment was associated with lower overall peripheral blood chimerism. IV transplants generally resulted in RF/LF ratios closer to 1, indicating that this method provided relatively uniform BM engraftment. This analysis is consistent with the graphical data in Fig. 3E and Supplementary Figure 2A, which shows that engraftment within injected and non-injected femurs was similar in IV transplanted mice.

Engraftment was always greater in injected femurs, and larger RF/LF ratios were more common in IF transplanted mice. The engraftment pattern in the spleens and non-injected femurs, relative to RF/LF ratios (Fig. 4B,C), was similar to the relative pattern observed for peripheral blood engraftment (Fig. 4A). This suggested that peripheral blood engraftment paralleled the engraftment patterns in the distal hematopoietic organs (LF and spleen). By contrast, no pattern was observed when the RF/LF ratio was plotted relative to hCD45 engraftment in the injected femur (Fig. 4D). This suggested that there was disconnect between engraftment in the injected femurs and engraftment in the distal organs.

The most striking data are enclosed within the “boxed” region of Fig. 4D, which identifies ~25% of mice with the highest levels of hCD45 engraftment within the injected femurs. All mice within this boxed region had RF/LF ratios >2. This implies that engraftment in the distal marrow of these animals was less than half of that observed within the injected marrow. In the most extreme case, the distal femur in one animal contained 10-fold fewer hCD45 cells than the injected femur. This observation is profound, as the injected femur in this animal was virtually saturated with hCD45 cells (>90% hCD45). These data suggest that saturation of the injected BM with human cells did not necessarily translate to high levels of systemic engraftment in the distal BM, spleen or peripheral blood. This engraftment phenomenon was only observed in animals that received IF transplants (Fig. 4D, red, green and blue data points), and was not observed in animals that received IV transplants (Fig. 4D, open circle data points).

## Discussion

Previous reports suggest that the efficacy of IF transplantation may be limited by the failure of injected cells to be retained within the injected marrow^17^. MSC spheroids are viable biological anchors that could be potentially used to retain donor HSC within the injected marrow. Assembling MSC into spheroids has been previously shown to enhance their secretion of HSC-supportive factors^19^,^20^. The enhanced supportive environment, coupled with anchorage, make MSC an ideal tool for CD34^+^ cell retention following IF transplant. Intriguingly, we found that anchoring CD34^+^ cells to MSC spheroids during IF transplantation compromised overall peripheral blood engraftment, relative to IF transplantation of CD34^+^ cells alone. This observation led us to carefully consider the hypothesis that anchorage could improve IF transplant outcomes, and asked the question: *does retention of donor CD34*^+^ *cells within the injected femurs actively compromise engraftment outcomes?*

While direct BM transplantation did not improve overall peripheral blood engraftment, greater local hCD45 and CD34 content was observed in injected femurs relative to distal femurs. Similar patterns favoring engraftment in the injected femur have been previously reported^8^,^12^,^17^,^21^. Based on this observation, it has been proposed that the high local engraftment efficiency makes IF transplantation an excellent tool for studying the engraftment of rare cell populations^8^,^12^. The greater number of primitive donor cells in femurs following IF transplantation has been interpreted as more efficient retention of primitive cells within the injected femur, coupled with a locally increased rate of self-renewal^17^. However, this interpretation ignores another possible hypothesis: the transient retention of donor cells in one bone marrow delivery site compromises their engraftment in distal marrow and other hematopoietic organs.

In most animal transplantation studies, animals are conditioned via irradiation. Irradiation liberates niche sites or “makes space” for donor HSC, and triggers a niche resident-cell signaling cascade that encourages homing of the donor cells^13^,^22^. The onset of this signaling cascade in murine bone marrow, and it’s tapering, occurs rapidly. There is an inflection point and tapering in the signaling that promotes homing as early as three days post irradiation^14^,^23^,^24^. Most laboratories synchronize these processes by conditioning animals and then transplanting donor cells 24–48 hours later. This window of opportunity is sufficient if cells are delivered via IV transplantation, as the majority of cells that will home to the murine marrow do so within 2–24 hours post infusion^13,25^.

The hypothesis that retention of donor cells within a single femur, following IF transplantation, will enhance transplant outcomes is contingent on the capacity for the locally engrafted population to subsequently disseminate throughout the recipient. However, the engraftment and dissemination potential of human HSC in a murine host will decrease as the host marrow recovers from irradiation. The engraftment capacity of donor hematopoietic cells is a function of both the irradiation dose, as well as the species relationship between the donor and host. Irradiation is an essential step if NSG mice are to be transplanted with human HSC, as otherwise human HSC are unable to compete with murine HSC for limited niche occupancy^21^. In a recent comparative study, human CD34^+^ cell engraftment potential was evaluated in sublethally irradiated and non-irradiated NSG mice^21^. At 12 weeks, peripheral blood hCD45 engraftment was ~60% and 5% for irradiated and non-irradiated mice, respectively. When murine HSC were not ‘displaced’ through irradiation, human HSC engraftment was reduced by approximately 10-fold. Similarly, as murine BM recovers from irradiation, human HSC engraftment efficiency declines. Thus, a risk associated with the retention of donor cells within one BM cavity, is that the distal BM recovers from conditioning and becomes less receptive to human donor cell repopulation.

Our data suggest that IF transplantation can saturate the injected femur, and yet may not contribute to high levels of systemic engraftment (boxed data in Fig. 4C). This observation highlights the tenuous balance between the benefits associated with avoiding donor cell loss to vascular non-hematopoietic organs, and the compromised outcomes that may result if the majority of donor cells are immediately retained within a localized BM site. A single murine femur represents only ~6% of the total BM volume^26^. Consequently, in this model, the benefits of improved localized engraftment are marginal if these cells cannot subsequently and efficiently repopulate distal niches. Previous data^8^ from John Dick’s laboratory aligns with our hypothesis that transient retention of donor cells within the injected femur leads to compromised engraftment at distal sites. In their study, clonal analysis was performed for engrafted human cells recovered from bones harvested from different regions in mice following direct bone marrow transplantation. Their data indicate that (1) a large number of clones populate the injected femurs, suggesting that many cells are retained within the injected femur; (2) many of the clones could also be found at distal sites, suggesting that clones could multiply in the injected femurs and then spread to distal sites; (3) some clones did not spread to distal sites, suggesting heterogeneity in migration capacity, or as we suggest - there is a temporal limitation in distal engraftment processes; and (4) some clones could be found at distal sites, but not in the injected site, suggesting that some IF injected cells might have leaked or migrated through the blood vessels to populate other sites.

While the previous work described above^8^ and our own study were performed using a short-term engraftment model (<16 weeks), a study from the laboratory of Connie Eaves, using a long-term engraftment model (27 weeks), demonstrated that the mobility of human HSC was substantially constrained in the BM of NSG mice^27^. At 27 weeks, the distribution of clones was not uniform between different BM sites of mice, when comparing the left hind leg, right hind leg and pelvic bones^27^. In one mouse, 32% of clones were found only in one of the three BM sites assessed, while in a second mouse, 53% of clones were found in only one BM site^27^. Earlier parabiont studies suggested that HSC flux between blood and BM occurred frequently and rapidly under homeostatic conditions, with 100–400 long-term engrafting HSC present in the circulation of mice at any given time^28^. Frequent recirculation and homing of human long-term HSC in a xenogeneic transplant model would be expected to produce a homogeneous engraftment pattern. However, previously published data suggest that human hematopoietic clones are not homogenously distributed throughout mice^8^,^27^. Our data suggest that the failure of human HSC to redistribute throughout the animal can result in both non-uniform spatial engraftment and compromised total cell engraftment. Like murine HSC^28^,^29^, it is likely that human HSC cyclically egress from niches and enter the circulation in mice; however, it appears that their capacity to re-engraft at distal BM sites is significantly impaired in NSG mice. Local BM retention of HSC, or their delayed distribution throughout the mouse following IF transplantation, can result in significantly compromised engraftment at distal sites.

Recent human clinical trials failed to deliver enhanced outcomes when allogeneic CB donor cells were delivered via direct marrow transplantation^11^. In these studies, a second CB unit was delivered via IV transplantation. It is possible that the greater access to distal niches enabled by IV transplantation provides a relative advantage in the allogeneic CB transplant setting. However, the underlying concept of localized retention of donor HSC in the BM may have merit in an autologous setting. Autologous HSC transplantation can be an effective treatment for non-Hodgkin’s lymphoma and multiple myeloma patients. In ~12% of patients, the number of HSC that can be mobilized is so low that this therapy is not viable^30^,^31^,^32^. A robust localized graft, established from a small number of autologous HSC may have significant therapeutic potential in this context. Because the graft is autologous, the factors that obstruct the competitive repopulation of human cells in the xenogeneic NSG model may not exist. Furthermore, IF transplantation may be ideal where genetic modification and engraftment of only a small population of HSC is necessary, for instance, to raise an immunological response to a cancer or virus^33^,^34^. In both cases, it would be more rational to use an autologous or syngeneic animal model and to ensure that spatial engraftment artifacts are considered during experimental design.

A further observation from our study was that co-administration of MSC with CD34^+^ cells did not appear to be improve hematopoietic engraftment. This is in contrast to previous studies, where results indicated that co-administration of MSC with CD34^+^ cells improved engraftment outcomes in NOD/SCID mice when delivered by IV^35^,^36^ and IF^37^. Our results parallel previous NSG studies, which reported no improvement in human CD34^+^ engraftment in the BM with co-administration of MSC^38^. We were unable to identify a study that directly compared co-administration of MSC in these two different mouse models (NOD/SCID vs NSG). Engraftment of human HSC in NOD/SCID mice is compromised by immune barriers such as the presence of murine natural killer cells^39^. The evolution of the NSG mouse reflects intensive research efforts to develop an animal model more tolerant of human xenografts, and increasingly, xenogeneic transplant studies are performed using the NSG mouse over other immunodeficient strains. The additional repression of the immune system in NSG mice results in 3.6-fold more sensitivity in detecting SCID repopulating cells than in NOD/SCID mice^39^. MSC are known to be immunomodulatory^40^, and others have proposed that their immunomodulatory activity might influence engraftment^41^. Thus the benefit of MSC^35^,^36^,^37^ may have been amplified in previous studies where the recipient NOD/SCID mice had a more active immune system. In our study, the immunomodulatory benefits of MSC did not appear to influence human HSC engraftment in the more immune compromised NSG mice.

In summary, we show that IF transplantation of human CD34^+^ cells enhanced local BM engraftment relative to distal hematopoietic sites in NSG mice. Engraftment at distal BM was compromised by the transient retention of donor cells within the injected femur, even in conditions that ultimately led to saturation of the local BM with human hematopoietic cells. We conclude that in this xenogeneic model, IF transplantation enhances local engraftment at the expense of engraftment at distal sites. We suggest that this artifact should be considered when utilizing IF transplantation in xenogeneic animal models.

## Materials and Methods

### Mice

NSG mice were purchased from the Jackson Laboratory^42^ and bred in the Animal Facility at the Translational Research Institute (TRI) in Brisbane. Adult mice (2–4 months) were sublethally irradiated with 250 cGy using a Gamma Cell 40 Caesium source 24 hours before human cell transplantation. The University of Queensland (UQ) and the Queensland University of Technology (QUT) Animal Ethics Committees authorized the animal procedures described here. All animal procedures were carried out in accordance with the approved guidelines (Ethics number: 1300000644).

### CD34^+^ cell isolation

CB was collected at the Mater Hospital in Brisbane following full-term births with the informed written consent from mothers. Ethics approval was granted by the Mater Health Services Human Research Ethics Committee and the Queensland University of Technology Human Ethics Committee, with all methods carried out in accordance with the approved guidelines (Ethics number: 1100000210). CD34^+^ cell isolation from CB was performed within 24 hours of collection. CB was diluted with PBS containing 2 mM EDTA and mononucleated cells were isolated by Ficoll-Paque (GE Healthcare) density centrifugation. CD34^+^ progenitor cells were purified using the CD34 MicroBead Kit-UltraPure and AutoMACS Pro Seperator (both from Miltenyi) as per manufacturer’s instructions. CD34^+^ cells were cryopreserved in 10% DMSO and 90% FBS, slowly frozen to −80 °C overnight and stored in liquid nitrogen until use. CD34^+^ cells were pooled from multiple CB donors to obtain sufficient cells for experiments. CD34-purity was assessed prior to cell culture by anti-human CD34 antibody (Miltenyi) staining and flow cytometry on the LSRII (Becton Dickinson), achieving more than 90% purity.

### MSC isolation and expansion

Human adult BM MSC isolation, culture and characterization were performed as previously described [14]. Ethics approval for aspirate collection was granted by the Mater Health Services Human Research Ethics Committee and the Queensland University of Technology Human Ethics Committee (Ethics number: 1000000938). Briefly, mononuclear cells were isolated from 20 ml of BM aspirates using density gradient centrifugation. MSC were enriched using plastic adherence overnight in a 20% O_2_ and 5% CO_2_ atmosphere at 37 °C in MSC expansion medium containing: low glucose DMEM (Life Technologies), 10% fetal bovine serum (FBS; Life Technologies), 10 ng/mL fibroblast growth factor-1 (FGF-1; Peprotech), and 100 U/ml penicillin/streptomycin (PenStrep; Life Technologies). After discarding the loose cells and replenishing the medium, MSC were further expanded in a 2% O_2_ and 5% CO_2_ atmosphere at 37 °C. Cells were passaged at 80% confluence using 0.25% trypsin/EDTA (Life Technologies) and new flasks were re-seeded at ~1,500 cells per cm^2^. MSC were used at passage 3. Using flow cytometry, cells were characterized for their expression of CD44, CD90, CD73, CD105, CD146, CD45, CD34, HLA-DR, and tri-lineage differentiation capacity, as described previously^43^.

### Cell culture preparation and mouse transplants

**Figure 1:**
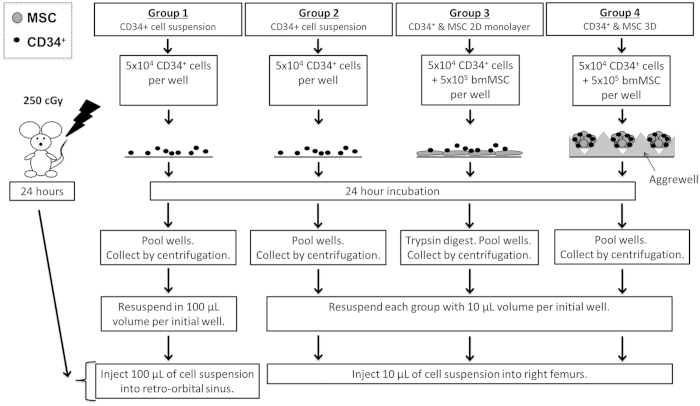
Schematic of experimental groups to test engraftment delivery strategies. Cultures were prepared as described schematically and injected in irradiated NSG mice via IV or IF transplants, 24-hours later.

**Figure 2:**
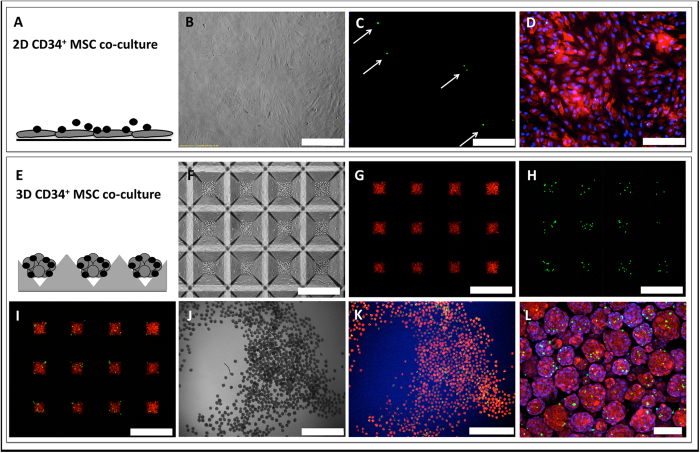
Images of 2D and 3D co-cultures. (**A–D**) 2D CD34^+^/MSC co-culture after 24 hours. (**B**) Phase contrast, (**C**) CD34^+^ cells labeled with CFSE (green), and (**D**) MSC labeled with CMPTX (red). Scale bars B–D = 400 μm. (**E–I**) 3D CD34^+^/MSC co-culture before (**F–I**) and after 24 hours (**J–L**). (**F**) Phase contrast image of microwells at time zero, (**G**) MSC labeled with CMPTX (red) time zero, (**H**) CD34^+^ cells labeled with CFSE (green) time zero, (**I**) overlay of labeled CD34^+^ and MSC at time zero; (**J**) Phase contrast of spheroids (4×) after 24 hour culture, (**K**) fluorescent image (4×), (**L**) 3D CD34^+^/MSC spheroids (confocal image, 20×). Scale bars (**F–I**) = 400 μm, Scale bar (**J,K**) = 1 mm, Scale bar (**L**) = 100 μm.

**Figure 3:**
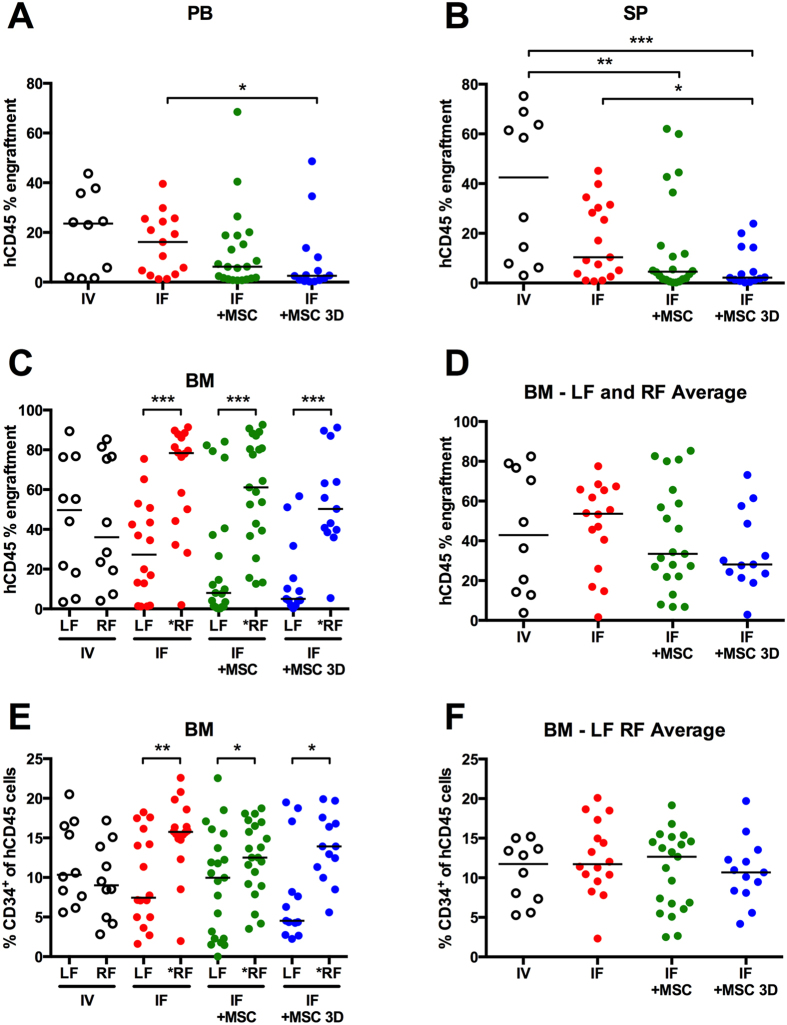
Human engraftment in peripheral blood (PB), spleen (SP), and bone marrow (BM) at 8 weeks. Cells in IV transplant were injected retro-orbitally, and in IF transplants, cells were injected into the right femur (RF; asterisk next to RF denotes “injected” femur). (**A**) hCD45 peripheral blood (PB) engraftment at 8 weeks. (**B**) hCD45 engraftment in spleens (SP). (**C**) hCD45 engraftment in both BM or each individual femur (LF and *RF). (**D**) Average LF and RF BM hCD45 engraftment. (**E**) hCD34 cell numbers in each femur (LF and *RF). (**F**) Average LF and RF BM hCD34 engraftment. (*P < 0.05, **P < 0.01, and ***P < 0.001).

**Figure 4:**
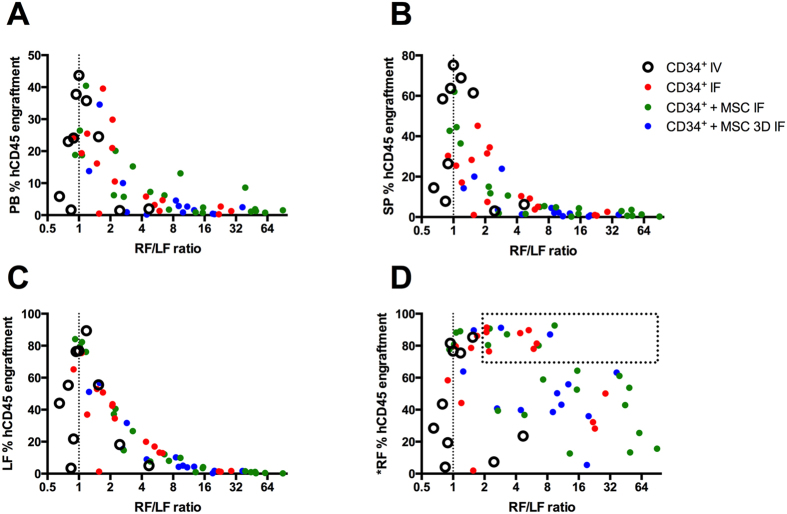
Relationship between hCD45 engraftment in specified tissues relative to the RF/LF ratio. RF/LF ratio is the hCD45 engraftment in the injected femur (RF) divided by the hCD45 engraftment in the non-injected femur (LF). (**A**) Graphical relationship between peripheral blood hCD45 engraftment and the RF/LF ratio. (**B**) Graphical relationship between spleen hCD45 engraftment and the RF/LF ratio. (**C**) Graphical relationship between the distal non-injected femur (LF) hCD45 engraftment and the RF/LF ratio. (**D**) Graphical relationship between hCD45 engraftment in the injected femur (*RF) and the RF/LF ratio.

The four different experimental groups assessed for human hematopoietic cell engraftment in NSG mice are represented schematically in Fig. 1. The four groups included: (1) IV delivery of CD34^+^ by retro-orbital injection; (2) direct marrow delivery of CD34^+^ by IF injection (3) co-delivery of a suspension of CD34^+^ and MSC by IF; and (4) co-delivery of CD34^+^ and MSC in 3D spheroids by IF. All cell cultures were established 24 hours prior to injecting mice. Cultures were prepared in 6-well plates with 5 × 10^4^ CD34^+^, with or without 5 × 10^5^ MSC, as indicated, in 4 mL of *X-Vivo* 15 media (Lonza) supplemented with 10 ng/ml of SCF (Amgen), TPO peptide (Auspep Pty Ltd.^44^), Flt-3 ligand (Miltenyi) and FGF-1. For groups 1, 2 and 3, cultures were initiated in 6-well tissue culture plates. The plates used for group 2 were additionally pre-coated with fibronectin (BD Biosciences) to allow for MSC attachment. Group 4 cultures were prepared in AggreWell 400Ex 6-well plates (StemCell Technologies) that were rinsed with 5% Pluronic-F127 (Sigma) to prevent cell adherence^45^. Since a single well of the AggreWell plate surface contained ~4,700 microwells, at the above cell seeding density used, ~4,700 spheroids were produced per well, consisting of ~110 MSC and ~10 CD34^+^ cells each.

Prior to cell injection in NSG mice, culture wells for each experimental group were pooled and concentrated. Group 2, which contained CD34^+^ cells and MSC adhered to the culture plate well, were first trypsinized to liberate the cells from the culture surface. NSG mice from each group were injected such that, each mouse received the equivalent of cells from a single well of the original 6-well plate, or 5 × 10^4^ CD34^+^ with or without 5 × 10^5^ MSC, as indicated. Cells were delivered in 100 μl of fresh *X-Vivo* 15 media for IV injections and in 10 μl for IF injections. For IV injections, mice were anaesthetized by isofluorane inhalation and cells injected in the retro-orbital sinus. For IF injections, mice were anaesthetized with 10 mg/ml ketamine/xylazine. A pilot hole was drilled through the joint into the right femur with a 26-guage needle, and the cells were injected with a 27-guage microsyringe needle (Hamilton).

### Flow cytometry quantification of human cell engraftment

Transplanted NSG mice were assessed after eight weeks for human hematopoietic cell engraftment. Mice were euthanized by CO_2_ asphyxiation and peripheral blood, femurs and spleen were harvested. PB was collected via cardiac puncture into heparin-containing tubes and lysed of red blood cells using lysis buffer (eBioscience) as per manufacturer’s instructions. Spleens were disrupted by grinding between two frosted glass cover slips. Left and right femurs from each mouse were crushed separately using a mortar and pestle. All tissue cell preparations were resuspended in cold 2% FBS/PBS and passed through a 40 μm cell strainer (Falcon). Approximately 10^6^ cells were used for antibody staining and flow cytometry analysis. Prior to antibody staining, cells were resuspended in blocking solution: mouse-Fc block (1 μg per sample) (BD Biosciences), 10% normal mouse serum (Life Technologies), and 1 mg/ml human IgG (Sigma) for 20 minutes at 4 °C. Fluorescence-conjugated antibodies were used to asses the level of human hematopoietic cell engraftment and lineage. Antibodies included human (h)CD45-VioBright FITC, hCD34-APC (both from Miltenyi), hCD3-APC_Cy7, hCD15-PE, hCD19-PE_Cy7, hCD33-BV421 (all from Biolegend), and mouse (m)CD45-APC (BD Biosciences). Cells were stained with the appropriate antibodies as recommended in the manufacturers’ instructions for 30 minutes at 4 °C. Dead cell exclusion was performed with the addition of 7-aminoactinomycin D (7-AAD; Life Technologies). Unstained cells, isotype controls and single colour controls were also prepared to facilitate gating and adjust for spectral overlap. Flow cytometry was performed on a LSRII (BD Biosciences). 50,000 to 100,000 total events were recorded. Data was analysed using FlowJo software (Tree Star). Percentage human engraftment was calculated as: % hCD45^+^ engraftment = hCD45^+^ cells/(hCD45^+^ cells + mCD45^+^ cells) × 100. Positive human engraftment was defined as more than 1% hCD45^+^ in one of the mouse tissues analysed. Non-engrafted mice were excluded from the analysis.

### Labeling of CD34^+^ cells and MSC for fluorescence imaging

CD34^+^ cells were stained using the CellTrace CFSE Cell Proliferation Kit (Life Technologies) and MSC were labelled with CellTracker Red CMTPX Dye as per manufacturer’s instructions, using a 5 μM working concentration of dye reagent in each case. CFSE-labelled CD34^+^ cells and CMTPX-labelled MSC were found to be 100% fluorescently labelled with the respective dye by flow cytometry. Cell cultures were prepared as described above. Monocultures and co-cultures were allowed to establish for 24 hours, at which point loose cells were gently washed away with 5× washes of 1 ml PBS. For 3D spheroid co-cultures, the cell aggregates were depleted of loose cells by washing them over a 35 μm cell strainer (Falcon). Cells were fixed with 4% paraformaldehyde (Sigma), stained with DAPI (Life Technologies). Confocal images of spheroids were captured with an Olympus FV1200 confocal microscope.

### Statistical Analysis

Statistical analysis was performed using Prism Version 5.0 (GraphPad). Differences between transplant groups were tested using the nonparametric Mann-Whitney test and differences between engraftment locations were tested using the Wilcoxon rank-sum test. Differences were considered to be significant for values of P < 0.05.

## Additional Information

**How to cite this article**: Futrega, K. *et al*. Direct bone marrow HSC transplantation enhances local engraftment at the expense of systemic engraftment in NSG mice. *Sci. Rep.* **6**, 23886; doi: 10.1038/srep23886 (2016).

## Supplementary Material

Supplementary Information
