# Supplementary material for: Direct bone marrow HSC transplantation enhances local engraftment at the expense of systemic engraftment in NSG mice
Source: Sci Rep. 2016 Apr 11;6:23886. doi: 10.1038/srep23886 (PMC4827391; doi:10.1038/srep23886)
Supplement: Supplementary Information [file srep23886-s1.pdf]

SUPPLEMENTARY FIGURE SECTION

Title: ***Direct bone marrow HSC transplantation enhances local engraftment at the expense of systemic engraftment in NSG mice***

Kathryn Futrega[1], William B Lott[1], and Michael R Doran[1,2*]

1. Queensland University of Technology (QUT) at the Translational Research Institute (TRI)
37 Kent Street, Brisbane, Queensland, Australia 4102

2. Mater Medical Research – University of Queensland at the Translational Research Institute (TRI)
37 Kent Street, Brisbane, Queensland, Australia 4102

***Email (corresponding author):** michael.doran@qut.edu.au

Figure Captions:

Supplementary Figure 1. MSC surface marker characterization and trilineage differentiation capacity. **(A)** BM-derived MSC were >95% positive for CD44, CD90, CD73, CD105, CD146, and <5% positive for CD45, CD34, and HLA-DR. Following 21 days of induction, MSC underwent **(B)** adipogenesis (oil red O staining of lipid vacuoles), **(C)** osteogenesis; alizarin red A staining of mineralized tissue, and **(D)** chondrogenesis; alcian blue staining of glycosaminoglycans. Scale bar = 400 μm.

Supplementary Figure 2. Representative flow cytometry gating for engraftment analysis in BM. **(A)** Forward scatter and side scatter, doublet exclusion and dead cell exclusion. **(B)** Human and murine CD45 identification (left panel), and isotype controls (right panel). **(C)** Hematopoietic lineage assessment of $CD19^+$ (B cells), $CD33^+$ (myeloid), $CD15^+$ (granulocytes), CD34 (progenitor), and CD3 (T-cells, not detected).

Supplementary Figure 3. Plots A-D show the relative hCD45 engraftment within injected femurs (*RF) and distal femurs (LF) for individual mice. A-D represent transplant groups 1-4 (see main text for details), respectively.

Supplementary Figure 4. Hematopoietic lineage commitment of hCD45 populations in the BM. (A-C) Lineage cells positive for $CD19^+$ (B cells), $CD33^+$ (myeloid), and $CD15^+$ (granulocytes). Lineage composition did not vary between femurs or between transplant groups.

Supplementary Figure 5. Hematopoietic lineage commitment of hCD45 populations in the peripheral blood (PB) and spleen (SP). (A-D) Lineage cells positive for $CD19^+$ (B cells), and $CD33^+$ (myeloid) cells. Lineage composition did not vary between transplant groups.

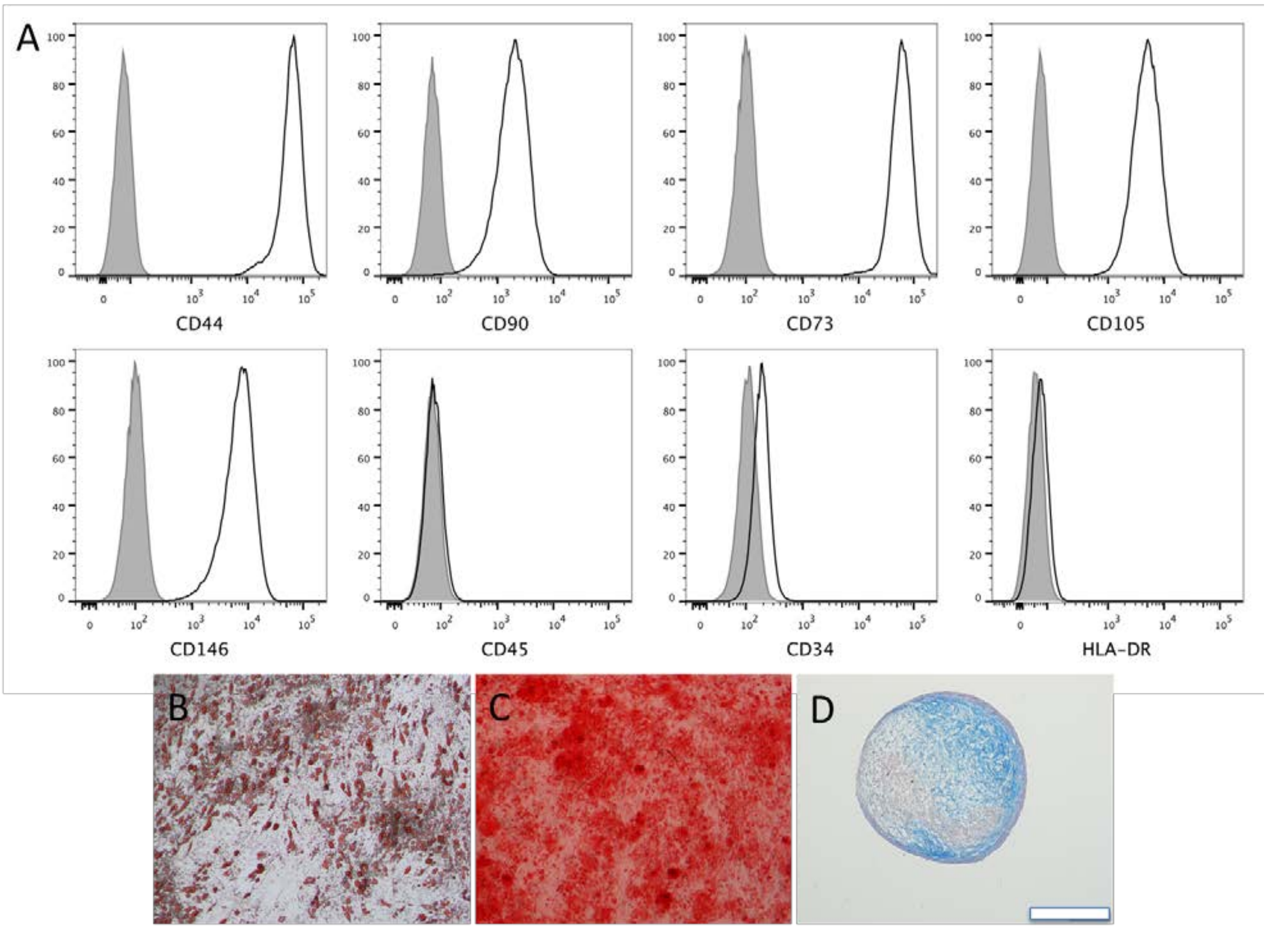

Supplementary Figure 1. MSC surface marker characterization and trilineage differentiation capacity. **(A)** BM-derived MSC were >95% positive for CD44, CD90, CD73, CD105, CD146, and <5% positive for CD45, CD34, and HLA-DR. Following 21 days of induction, MSC underwent **(B)** adipogenesis (oil red O staining of lipid vacuoles), **(C)** osteogenesis; alizarin red A staining of mineralized tissue, and **(D)** chondrogenesis; alcian blue staining of glycosaminoglycans. Scale bar = 400 μm.

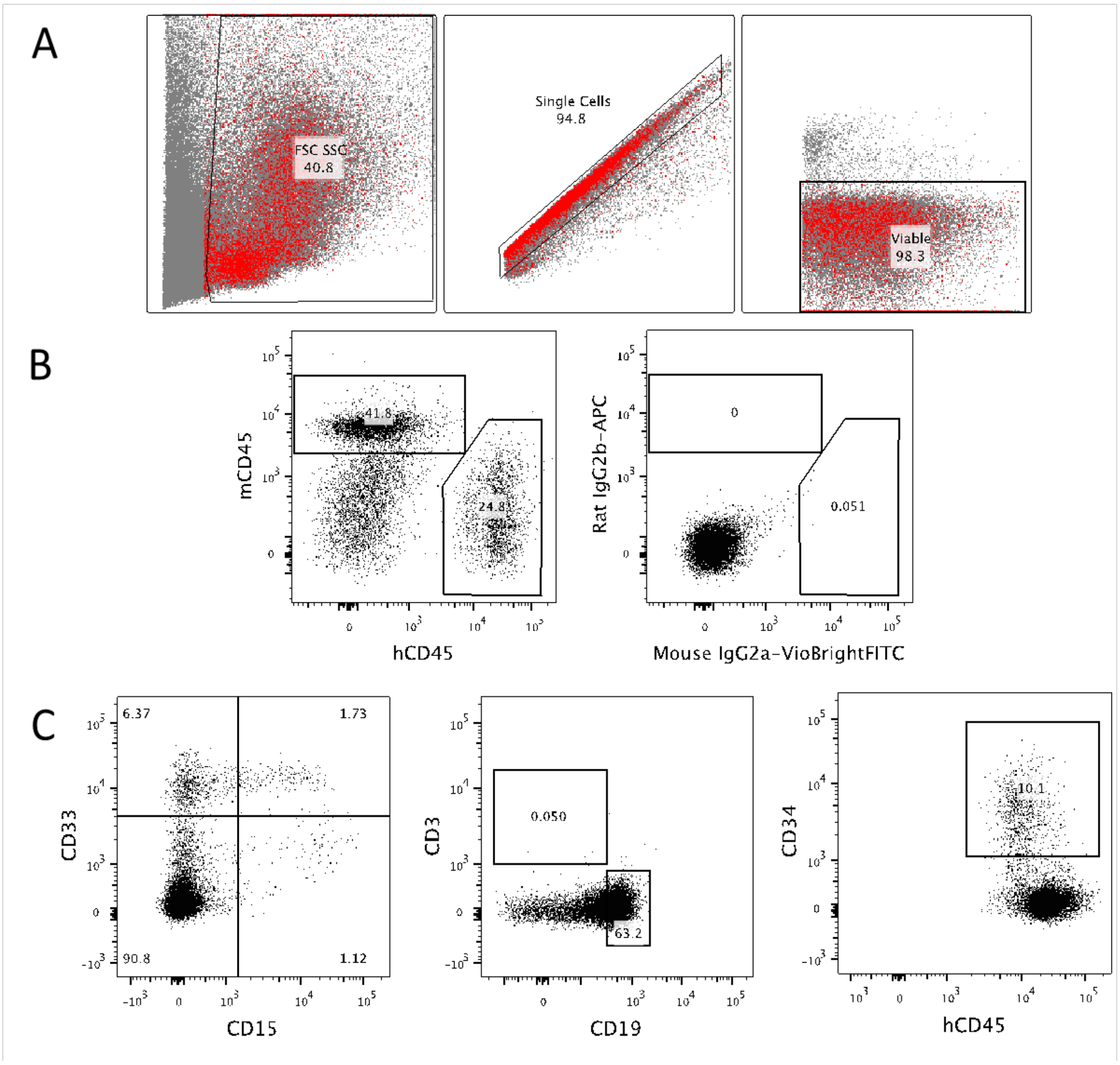

Supplementary Figure 2. Representative flow cytometry gating for engraftment analysis in BM. **(A)** Forward scatter and side scatter, doublet exclusion and dead cell exclusion. **(B)** Human and murine CD45 identification (left panel), and isotype controls (right panel). **(C)** Hematopoietic lineage assessment of CD19$^+$ (B cells), CD33$^+$ (myeloid), CD15$^+$ (granulocytes), CD34 (progenitor), and CD3 (T-cells, not detected).

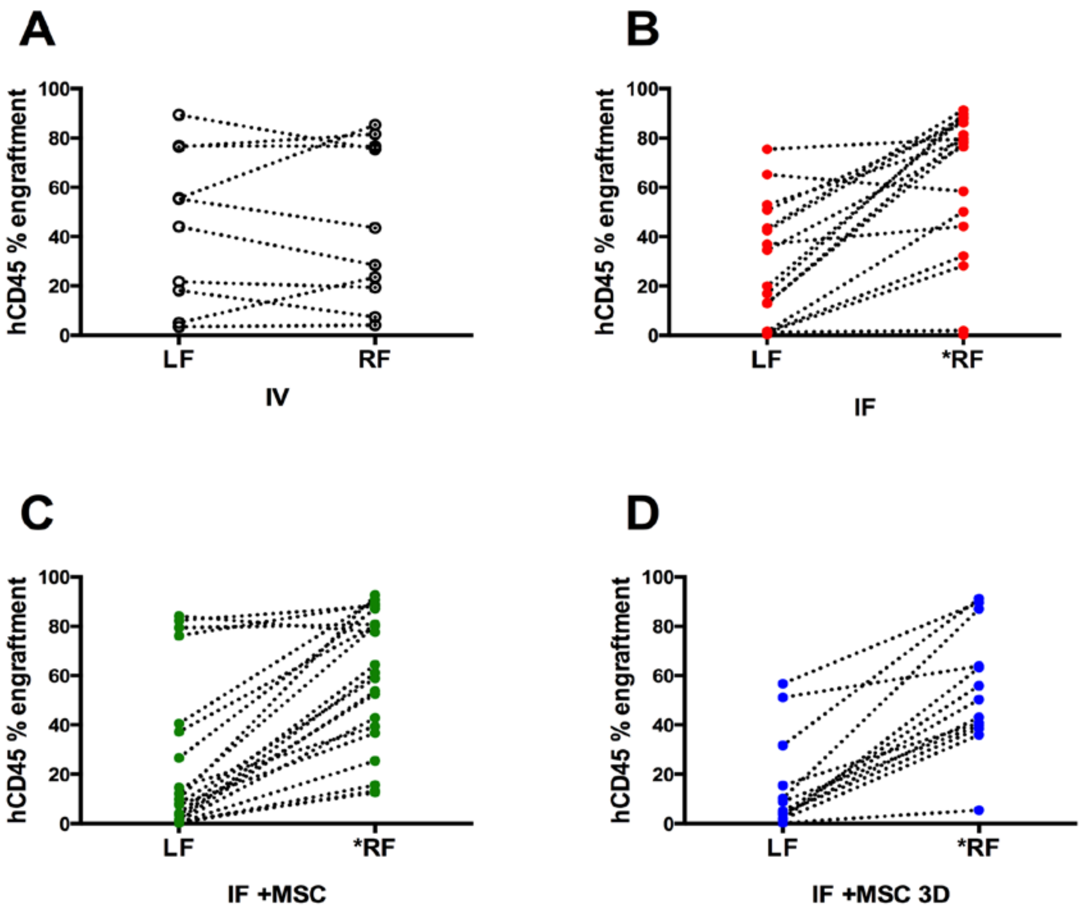

Supplementary Figure 3. Plots A-D show the relative hCD45 engraftment within injected femurs (*RF) and distal femurs (LF) for individual mice. A-D represent transplant groups 1-4 (see main text for details), respectively.

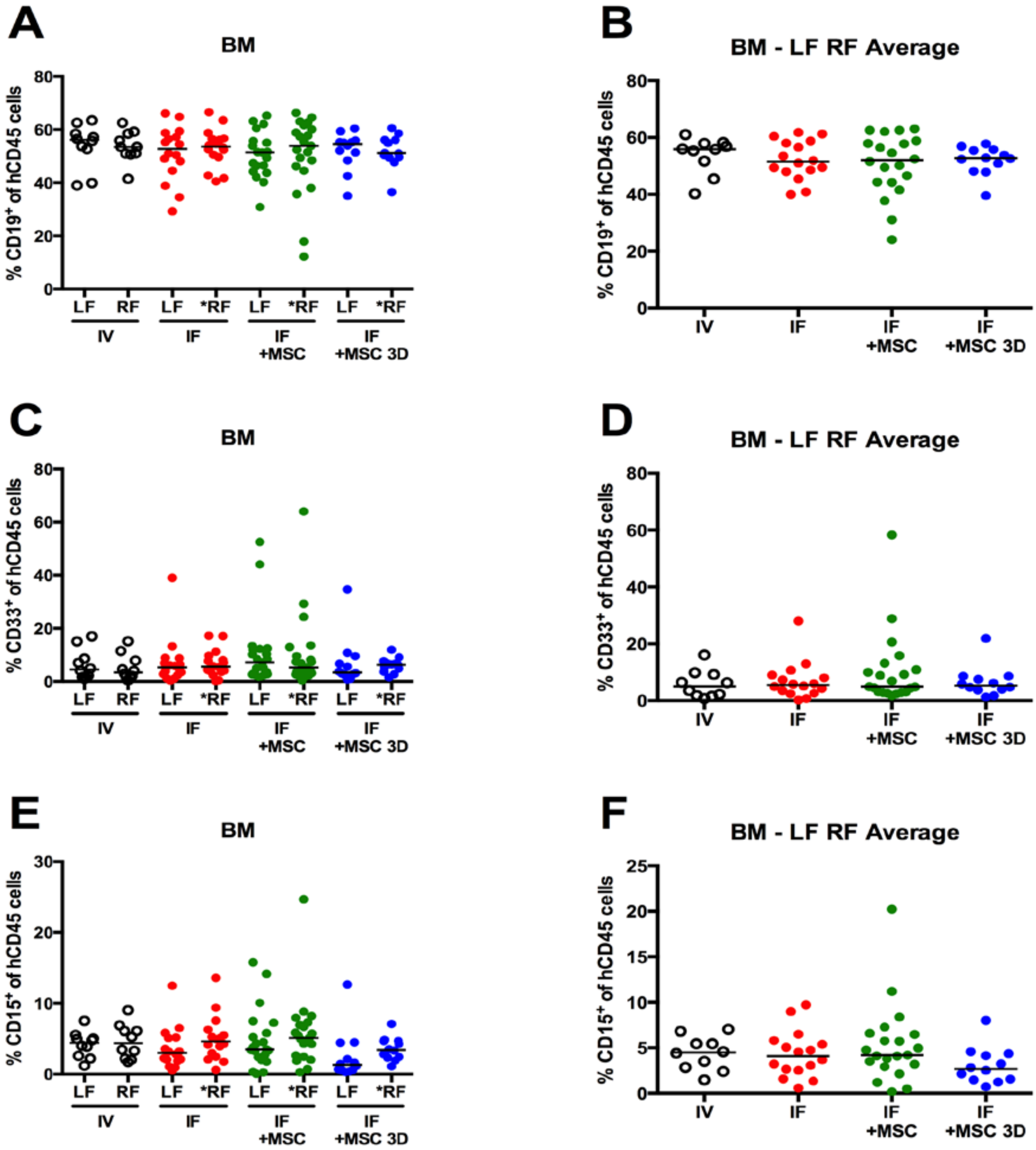

Supplementary Figure 4. Hematopoietic lineage commitment of hCD45 populations in the BM. (A-C) Lineage cells positive for $CD19^+$ (B cells), $CD33^+$ (myeloid), and $CD15^+$ (granulocytes). Lineage composition did not vary between femurs or between transplant groups.

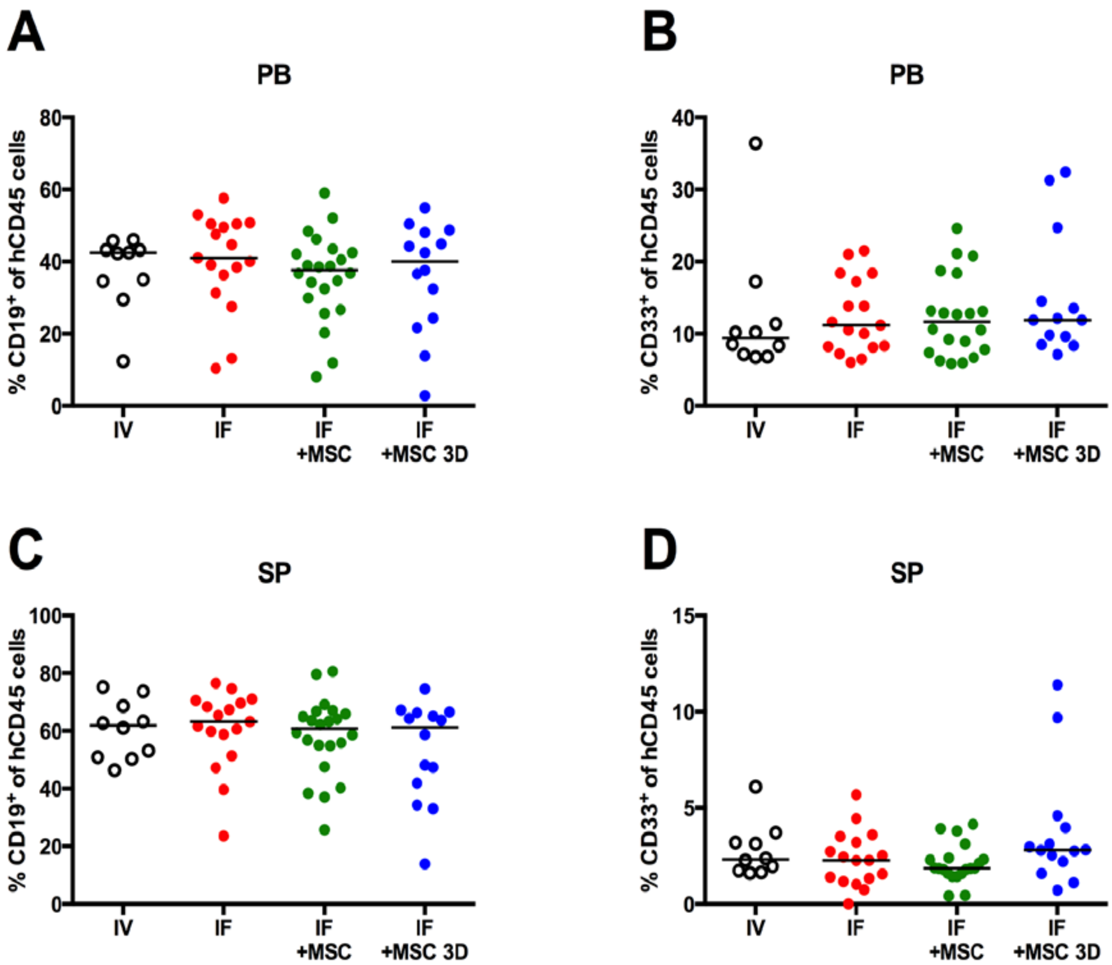

Supplementary Figure 5. Hematopoietic lineage commitment of hCD45 populations in the peripheral blood (PB) and spleen (SP). (A-D) Lineage cells positive for $CD19^+$ (B cells), and $CD33^+$ (myeloid) cells. Lineage composition did not vary between transplant groups.
